# Prevention of Radiation-Induced Salivary Gland Dysfunction Utilizing a CDK Inhibitor in a Mouse Model

**DOI:** 10.1371/journal.pone.0051363

**Published:** 2012-12-07

**Authors:** Katie L. Martin, Grace A. Hill, Rob R. Klein, Deborah G. Arnett, Randy Burd, Kirsten H. Limesand

**Affiliations:** 1 Physiological Sciences Graduate Interdisciplinary Program, University of Arizona, Tucson, Arizona, United States of America; 2 Department of Pathology, University of Arizona, Tucson, Arizona, United States of America; 3 Department of Nutritional Sciences, University of Arizona, Tucson, Arizona, United States of America; National Taiwan University, Taiwan

## Abstract

**Background:**

Treatment of head and neck cancer with radiation often results in damage to surrounding normal tissues such as salivary glands. Permanent loss of function in the salivary glands often leads patients to discontinue treatment due to incapacitating side effects. It has previously been shown that IGF-1 suppresses radiation-induced apoptosis and enhances G2/M arrest leading to preservation of salivary gland function. In an effort to recapitulate the effects of IGF-1, as well as increase the likelihood of translating these findings to the clinic, the small molecule therapeutic Roscovitine, is being tested. Roscovitine is a cyclin-dependent kinase inhibitor that acts to transiently inhibit cell cycle progression and allow for DNA repair in damaged tissues.

**Methodology/Principal Findings:**

Treatment with Roscovitine prior to irradiation induced a significant increase in the percentage of cells in the G**_2_**/M phase, as demonstrated by flow cytometry. In contrast, mice treated with radiation exhibit no differences in the percentage of cells in G**_2_**/M when compared to unirradiated controls. Similar to previous studies utilizing IGF-1, pretreatment with Roscovitine leads to a significant up-regulation of p21 expression and a significant decrease in the number of PCNA positive cells. Radiation treatment leads to a significant increase in activated caspase-3 positive salivary acinar cells, which is suppressed by pretreatment with Roscovitine. Administration of Roscovitine prior to targeted head and neck irradiation preserves normal tissue function in mouse parotid salivary glands, both acutely and chronically, as measured by salivary output.

**Conclusions/Significance:**

These studies suggest that induction of transient G**_2_**/M cell cycle arrest by Roscovitine allows for suppression of apoptosis, thus preserving normal salivary function following targeted head and neck irradiation. This could have an important clinical impact by preventing the negative side effects of radiation therapy in surrounding normal tissues.

## Introduction

It is estimated that over 40,000 cases of head and neck cancer will be diagnosed in the United States in 2012 [1]. Head and neck cancer is the 6^th^ most common in the world, having a higher incidence in developing countries [2]. These cancers are often associated with increased tobacco and alcohol use [2]. The standard of care for these cancers includes surgical resection of the tumor and a combination of chemotherapy and ionizing radiation. However, irradiation of the head and neck region often exposes surrounding non-diseased tissues to incidental radiation, resulting in secondary side effects. Intensity-modulated radiation therapy (IMRT) is a type of radiotherapy used to spare normal tissues, like the salivary glands, in order to reduce the secondary side effects [3]. IMRT has made improvements in salivary gland sparing; however, depending on tumor location and grade, radiation-induced damage to the salivary glands still occurs resulting in salivary gland dysfunction.

Dysfunction of the salivary glands following radiation occurs in two stages, acute and chronic. Clinically, acute salivary gland dysfunction occurs within days and is characterized by loss of salivary flow, loss of acinar cells, glandular shrinkage, and changes in saliva composition. Chronic salivary gland dysfunction occurs months to years following radiotherapy and is characterized by reduced salivary flow and changes in saliva composition [4]. Affected patients suffer from xerostomia (dry mouth), oral mucositis, difficulty speaking, increased oral pathologies, difficulty chewing and swallowing food, as well as malnutrition due to loss of salivary flow [4]. Due to the dysfunction of the salivary glands, patients must resort to temporary treatments for xerostomia to maintain adequate nutrition and hydration. Overall there is a significant reduction in quality of life for those undergoing treatment.

The mechanisms responsible for the elevated radiosensitivity of salivary glands are not well understood [4]. Typically radiosensitive tissues are relatively undifferentiated with a high level of proliferation [5]. In contrast, salivary glands are highly differentiated tissues with very low levels of proliferation. Therefore, the response of salivary glands to radiation exposure could serve as a model for other normal differentiated tissues in close proximity to other cancers. In addition, radiosensitivity of normal tissues is highly dependent on the activity of wild type p53 and a number of tumors have mutated or altered p53 activity [6]. Theoretically this may provide an important therapeutic window, as the response of tumors that are highly proliferative with modulated p53 activity is likely to be quite different from differentiated normal tissues with unaltered p53 activity.

In mouse models, loss of salivary gland function has been shown to be highly correlated with radiation-induced apoptosis of salivary acinar cells [4]. Previous studies focused on IGF-1 and its known ability to suppress apoptosis by activating endogenous Akt [4], [7]–[9]. A study by Mitchell et al. showed that parotid glands of mice exhibited increased G**_2_**/M arrest when treated with IGF-1 prior to head and neck irradiation. In contrast, parotid glands treated with radiation alone showed no induction of cell cycle arrest at G**_2_**/M, which may serve as a potential mechanism for the relative radiosensitivity of this tissue. Furthermore, IGF-1 was found to cause sustained p21 expression levels, increase inhibitory phosphorylation of cdk1 (tyrosine^15^) and decrease protein levels of cdc25A [10]. The cell cycle arrest that is demonstrated in irradiated mice pre-treated with IGF-1 corresponds with decreased apoptosis and normal salivary function.

In an effort to recapitulate the findings of IGF-1 without the potential adverse effects of a growth factor, Roscovitine, an inhibitor of the cell cycle, was investigated. Roscovitine acts to transiently arrest the cell cycle at the G**_2_**/M phase by competing for the ATP binding site in the catalytic cleft of the cyclin-dependent kinase [11]. Additionally, Roscovitine treatment exhibits direct inhibition specificity for CDK2, CDK7, and CDK9, as well as indirect inhibition of CDK1 [11], [12]. In this study we demonstrate that parotid glands of mice pretreated with Roscovitine prior to targeted head and neck irradiation exhibit cell cycle arrest at the G**_2_**/M phase. We also demonstrate that Roscovitine treatment leads to upregulation of p21, which is necessary for maintenance of cell cycle arrest. Additionally, we show that irradiated mice pretreated with Roscovitine have salivary function similar to unirradiated controls, making it a clinically translatable small molecule therapeutic for use in preservation of salivary gland function.

## Materials and Methods

### Mice

Experiments were conducted with 4 to 6 week old female FVB mice (Taconic, Oxnard, CA). All mice were housed and treated according to protocols approved by the University of Arizona Institutional Animal Care and Use committee (#08–090 and #11–282). All mice were observed to ensure weight loss following radiation did not exceed 10%.

### Treatment

Mice were treated with radiation, intraperitoneal injections of Roscovitine, or a combination of the two. R-roscovitine was obtained from EMD Millipore Chemicals (Billerica, MA) or Cyclacel Limited (Dundee, UK) and was reconstituted in DMSO (Fisher Scientific Pittsburg, PA). For irradiation treatments, mice were anesthetized with intramuscular injections of ketamine/xylazine (50 mg/kg/10 mg/ml) (Western Medical Supply, Arcadia, CA). Once anesthetized, mice were placed individually in a holding device, allowing exposure of the head and neck region to radiation while the rest of the body was shielded with >6 mm lead. Experiments with complete shielding (>6 mm lead) did not induce significant loss of salivary gland function. The mice were exposed to a 5Gy single dose of targeted head and neck irradiation (^60^Co therapeutic irradiator, Theratron-80, Atomic Energy of Canada Ltd., Ottawa, Canada). To ensure consistent dosing, a time calculation was performed prior to each irradiation, taking into account source output. The Experimental Radiation Shared Service (ERSS) confirms the proper dose (dosimetry) through routine calibration checks. Mice treated with Roscovitine received a single intraperitoneal injection of either 25 mg/kg or 100 mg/kg dose. For mice that received a combination treatment, Roscovitine was administered 2 hours prior to irradiation. Irradiated and unirradiated control animals received vehicle (DMSO) injections 2 hours prior to irradiation. For fractionated radiation treatment, mice were treated with 2 Gy/day for five consecutive days and mice receiving combination treatment were injected with Roscovitine 2 hours prior to each radiation.

### Flow Cytometry

Pairs of parotid glands were minced in dispersion media of 1 mg/ml Hyaluronidase (Sigma-Aldrich, St. Louis, MO), 1 mg/ml Collagenase P (Roche Diagnostics, Indianapolis, IN), in Modified Hanks Solution mixed at 37°C for 20 minutes, re-suspended with 1µM EGTA (Fisher Scientific), and further mixed at 37°C for 10 minutes. Tissue was disrupted using wide-orifice pipette tips and filtered into centrifuge tubes through 40µm filter caps. This single cell suspension was centrifuged 2× in cold PBS and fixed by adding 500 µL of cold 100% ethanol. Cells were stored at −20°C overnight, centrifuged, then re-suspended in 462.5 µL of cold PBS. Cells were incubated at 37°C for 30 minutes following addition of 25 µL of RNase A (Qiagen, Valencia, CA) and 12.5 µL of propidium iodide (MP Biomedicals, Solon, OH). The cell cycle distribution was measured by the AZCC/ARL Division of Biotechnology Cytometry Core Facility using a FACScan flow cytometer.

### RNA Isolation and RT-PCR

Mouse parotid glands were removed and placed in RNAlater (Qiagen) and RNA was isolated following manufacturer’s instructions. 1 µg of RNA was reverse transcribed into cDNA using the Superscript III Kit as described by the manufacturer (Invitrogen, Grand Island, NY) and diluted 1∶5 for subsequent analysis by real-time PCR.

### Real-Time PCR

A real-time reaction mix was prepared from 5 µl of diluted cDNA, 1 µl of mixed forward and reverse primers at 10 µM each, 12.5 µl SYBR Green (Qiagen), and nuclease-free water to a final volume of 25 µl. The following primers were purchased from IDT (San Diego, CA): p21 forward 5′-GCC ACA GCG ACC ATG TCC AA-3′, p21 reverse 5′-GCG TCT CCG TGA CGA AGT CAA A-3′, S15 forward 5′-ATC ATT CTG CCC GAG ATG GTG-3′, S15 reverse 5′-TGC TTT ACG GGC TTG TAG GTG-3′. Real-time PCR was conducted in triplicate for each cDNA sample using an iQ5 Real-Time PCR Detection System (Bio-Rad, Hercules, CA). Forty cycles of PCR were performed (95°C for 15 seconds, 54°C for 30 seconds, 72°C for 30 seconds) with fluorescence detection occurring during the 72°C step at each cycle. The data were analyzed using a 2^−ΔΔCt^ method [13]. The results were normalized to S15, a gene that is unchanged with treatment. The normalized values were plotted as relative fold over untreated.

### Western Blotting

Parotid glands were homogenized in RIPA buffer with SIGMAFAST protease inhibitor cocktail (Sigma-Aldrich) and 5 mM sodium orthovanadate (Fisher Scientific) then 100 mM PMSF was added (Pierce/Thermo Scientific, Rockford, IL). Samples were boiled and sonicated until homogenous. Coomasie Plus Protein Assay Reagent (Thermo Scientific) was used to determine protein concentrations. 100 µg of each sample was loaded on 10% or 12% polyacrylamide gels, transferred to a 0.45 µm Immobilon-P membrane (Millipore, Billica, MA) blocked with milk, and immunoblotted with one of the following antibodies: anti-ERK (Promega, Madison, WI), anti-p21 (Abcam, Cambridge, MA), pMDM2 (Cell Signaling Technology, Danvers, MA), MDM2 (Oncogene Research Products, San Diego, CA), pAKT (Cell Signaling Technology), AKT (Cell Signaling Technology). Secondary antibodies were conjugated with HRP and ECL substrate (Pierce/Thermo Scientific) was used for detection as instructed by manufacturer. Membranes were stripped using restore western blotting stripping buffer (Fisher Scientific), blocked and probed as described above.

### PCNA Staining

Glands were fixed overnight with 10% formalin (Fisher Scientific), transferred to 70% ethanol, then paraffin embedded. Tissues were cut into 4 µm sections by the Histology Service Laboratory in the Department of Cellular and Molecular Medicine at the University of Arizona. Slides were incubated at 37°C for 30 minutes and rehydrated in histoclear (National Diagnostics, Atlanta, GA), alcohol gradations, and distilled water. Nonspecific peroxidase activity was neutralized with 3% H_2_O_2_ (Fisher Scientific) and antigen retrieval was achieved by microwaving slides in citrate buffer (pH 6.0) twice for 5 minutes then cooled for 20 minutes. Slides were washed then blocked with ABC Rabbit Kit (Vector Laboratories, Burlingame, CA). Slides were incubated overnight at 4°C in PCNA primary antibody (Santa Cruz Biotechnology). Slides were washed and incubated in secondary antibody. DAB (Biogenex Laboratories, Fremont, CA) incubation occurred for 6–8 minutes to allow for color development. Slides were then counterstained using Gill’s hematoxylin (Sigma-Aldrich), dehydrated, and mounted with Protocol Securemount (Fisher Scientific). Images were taken using Leica DM5500 and 4 megapixel Pursuit camera. PCNA-positive acinar cells in parotid sections were counted from a minimum of 3 images per slide at 200x, with 3–4 mice per treatment group.

### Caspase-3 Staining

Glands were fixed overnight with 10% formalin (Fisher Scientific), transferred to 70% ethanol, then paraffin embedded. Tissues were cut into 4 µm sections by the Histology Service Laboratory in the Department of Cell Biology and Anatomy at the University of Arizona. Slides were incubated at 37°C for 45 minutes and rehydrated in histoclear (National Diagnostics), alcohol gradations, and distilled water. Antigen retrieval was achieved by microwaving slides in citrate buffer (pH 6.0) twice for 5 minutes then cooled for 20 minutes. Slides were washed then blocked with ABC Rabbit Kit (Vector Laboratories). Slides were incubated overnight at 4°C in activated caspase-3 primary antibody (Cell Signaling Technology). Slides were washed and nonspecific peroxidase activity was neutralized with 1% H_2_O_2_ (Fischer Scientific). Following secondary antibody incubation, DAB (Biogenex Laboratories) incubation occurred for 2–6 minutes to allow for color development. Slides were counterstained using Gill’s hematoxylin (Sigma-Aldrich), dehydrated, and mounted with Protocol Securemount (Fisher Scientific). Images were taken using Leica DM5500 and 4 megapixel Pursuit camera. Caspase-3 positive acinar cells in parotid sections were counted from a minimum of 5 images per slide at 400x, with 3–4 mice per treatment group.

### Saliva Collections

Stimulated saliva collections were performed on female FVB mice at 3, 14, and 30 days following radiation treatment. Mice received an intraperitoneal injection of carbachol at 0.25 mg/kg (Sigma-Aldrich) immediately prior to collection. Mice were placed into a restraint device and saliva was collected for 5 minutes via vacuum aspiration into pre-weighed tubes kept on ice. Each treatment group contains a minimum of 8 mice.

### Statistics

Flow cytometry data were analyzed by a one-way analysis of variance (ANOVA), followed by a post-hoc Student-Newman-Keuls analysis. Real time RT-PCR data, PCNA data, and Caspase-3 data were analyzed by a one-way analysis of variance (ANOVA), followed by a post-hoc Tukey-Kramer multiple comparison test. Salivary flow rates were normalized to the respective untreated groups for days 3, 14, and 30, and then analyzed by ANOVA followed by post-hoc Student-Newman-Keuls analysis. Statistical analysis of data was done using Graph-Pad software (version 5.0, San Diego, CA) and graphical generation was done using Microsoft Excel.

## Results

### Increased Cell Cycle Arrest in Irradiated Parotid Glands Pretreated with Roscovitine

**Figure 1:**
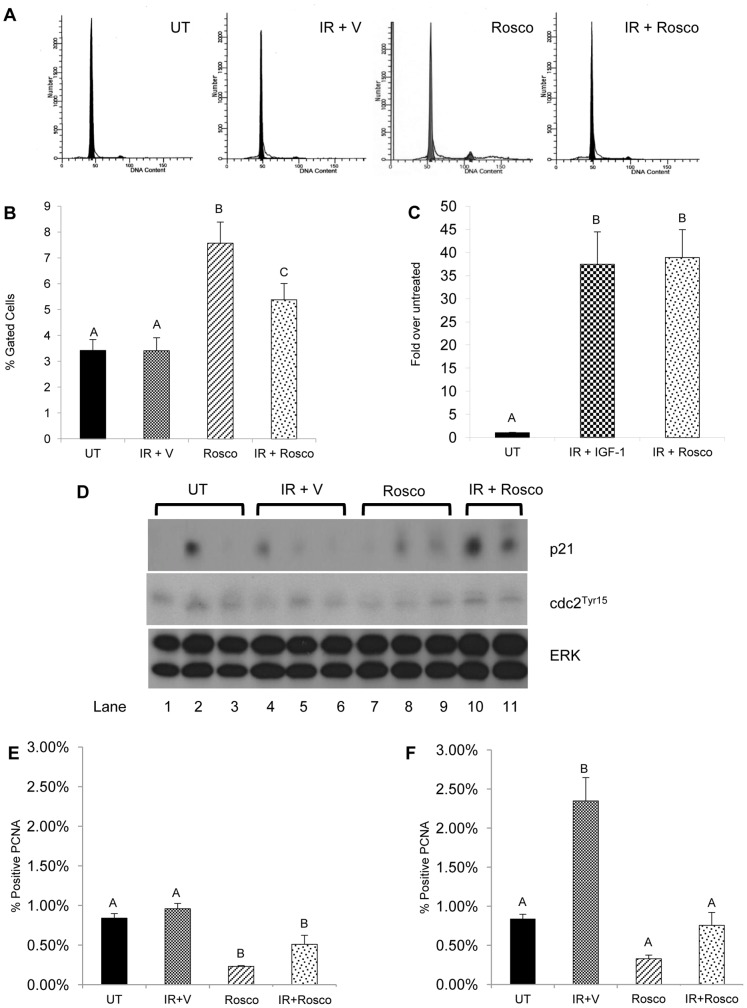
Increased cell cycle arrest in irradiated parotid glands pretreated with Roscovitine. The head and neck region of female FVB mice was treated with a 5Gy dose of radiation with or without 100 mg/kg Roscovitine pretreatment. Parotid glands were removed 6 hours after radiation treatment. A) Representative flow cytometry histograms from untreated (UT), irradiated with vehicle pre-treatment (IR+V), Roscovitine alone (Rosco), and Roscovitine prior to irradiation (IR+Rosco). B) Tissues were dispersed, stained with propidium iodide, and analyzed by flow cytometry. Graphical representation of the mean percentage of cells gated in G2/M with SEM from ≥9 mice per treatment. C) RNA was isolated from parotid glands, treated as stated above and real-time RT-PCR was run with primers for p21 amplification. Results were calculated using the 2^−ΔΔCt^, normalized to untreated and displayed as mean with SEM≥4 mice per treatment. D) Parotid tissues were treated as stated above and collected 6 hours following radiation for immunoblotting and prepared as described in the materials and methods, and membranes were probed for p21 (top panel) and cdc2^Tyr15^ (middle panel), with total ERK (bottom panel) as a loading control. Tissues were collected at 24 hours (E) and 48 hours (F) from irradiated mice pretreated with Roscovitine, embedded in paraffin, and stained for PCNA as described in the materials and methods. Results are graphed as the number of PCNA-positive acinar cells as a percentage of total counted acinar cells. The data are shown as the mean with SEM≥3 mice per treatment group. Treatment groups with the same letters are not statistically different from each other.

We have previously shown an accumulation of cells in the G**_2_**/M phase in irradiated salivary glands pre-treated with IGF-1 [10]. This corresponds with transactivation of genes involved in cell cycle arrest following activation of p53 by radiation-induced DNA damage [14]. To test the ability of Roscovitine to arrest the cell cycle at the G**_2_**/M phase, a single cell suspension from irradiated salivary glands pre-treated with Roscovitine was stained with propidium iodide and analyzed by flow cytometry. Representative flow cytometry histograms are shown in Figure 1A. Radiation treatment does not increase the percentage of cells in G**_2_**/M six hours after treatment (Figure 1B). In contrast, irradiated parotid glands pretreated with Roscovitine show a significant increase in the percentage of cells in G**_2_**/M when compared to untreated and irradiated populations (p<0.05). Roscovitine alone also induces cell cycle arrest at G**_2_**/M, as shown by the increased percentage of cells in G**_2_**/M (p<0.001).

G**_2_**/M arrest induced by DNA-damage is maintained by p21, an inhibitor of many cyclin dependent kinases including cdk1 and cdk2 [15]–[17]. Previously it was demonstrated that irradiated parotid glands pre-treated with IGF-1 showed sustained expression of p21, thus corresponding to increased cellular accumulation in G**_2_**/M [10]. To confirm maintenance of G**_2_**/M cell cycle arrest, real-time RT-PCR was used to determine expression levels of p21 in irradiated parotid glands that were pretreated with Roscovitine or IGF-1 (Figure 1C). There is significantly higher expression of p21 in parotid glands six hours post radiation treatment when pretreated with either Roscovitine or IGF-1 (p<0.01). This finding corresponds with increased p21 protein levels and increased inhibitory phosphorylation of cdc2 (tyrosine^15^) found in irradiated parotid glands pretreated with Roscovitine (Figure 1D, lanes 10–11).

To confirm the ability of Roscovitine to induce cell cycle arrest in irradiated parotid glands, proliferation was measured by staining the tissue with proliferating cell nuclear antigen (PCNA). As the salivary glands are a highly differentiated, low proliferating tissue [4], very few positively stained PCNA cells are observed in untreated tissue (Figure 1E). After 24 hours, the percentage of PCNA positive cells in tissues treated with radiation is unchanged from unirradiated levels. In irradiated tissues pretreated with Roscovitine, there is a significant decrease in the percentage of PCNA positive cells when compared to radiation (p<0.001). This decrease in proliferation is transient as irradiated Roscovitine treated salivary glands are not significantly different from unirradiated controls 48 hours post treatment. In contrast, there is a significant increase in the number of positive PCNA cells (p<0.01) in irradiated salivary glands at the 48 hour time point. Salivary gland tissues treated with Roscovitine alone have a significant decrease (p<0.01) in the number of PCNA positive cells at 24 hrs which returns to unirradiated levels by 48 hours. Taken together, these results suggest Roscovitine induces cell cycle arrest in normal salivary glands exposed to radiation treatment.

### Reduced Apoptosis in Irradiated Parotid Glands Pretreated with Roscovitine

**Figure 2:**
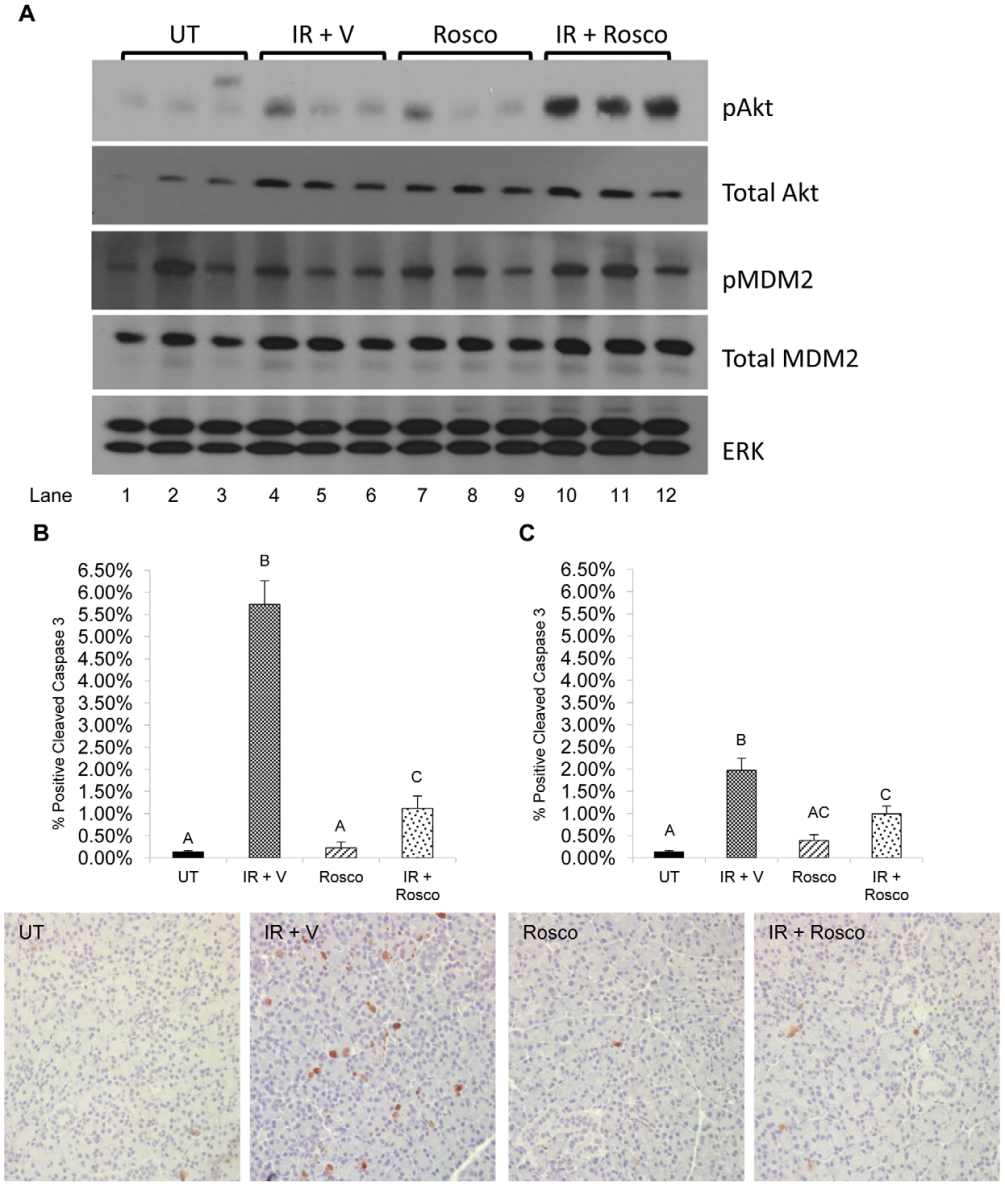
Reduced apoptosis in irradiated parotid glands pretreated with Roscovitine. Mice were treated as stated in Figure 1. A) Parotid glands were removed 6 hours after radiation treatment for immunoblotting, prepared as described in the materials and methods, and membranes were probed for phosphorylated Akt, total Akt, phosphorylated MDM2, and total MDM2. All membranes were striped and re-probed for total ERK as a loading control. Images are representative of several experiments. Abbreviations represent untreated (UT), irradiated with vehicle pre-treatment (IR+V), Roscovitine alone (Rosco), and Roscovitine prior to irradiation (IR+Rosco). Tissues were collected at 24 hours (B) and 48 hours (C), embedded in paraffin, and stained for cleaved caspase-3 as described in the materials and methods. Results are displayed as the number of cleaved caspase-3 positive cells as a percentage of total counted cells. The data are graphed as the mean with SEM≥3 mice per treatment group. Representative images of cleaved caspase-3 staining are shown below the graphs. Treatment groups with the same letters are not statistically different from each other.

It has previously been demonstrated that IGF-1 activates Akt leading to suppression of apoptosis in irradiated salivary acinar cells [7], [8]. To investigate this pathway in irradiated parotid glands pretreated with Roscovitine, endogenous protein levels were measured using immunoblotting. Roscovitine causes increased phosphorylation of Akt and MDM2, an Akt substrate that reduces p53 levels through its ubiquitin ligase function (Figure 2A, lanes 10–12). To evaluate the effects of activating this pathway on cell death, tissues were stained for cleaved caspase-3, a marker of apoptosis (graphed in Figure 2B and representative images below). There is a significantly higher percentage of cleaved caspase-3 positive cells with radiation treatment when compared to unirradiated controls (p<0.001). In irradiated parotid glands pretreated with Roscovitine, there is a significant decrease in the percentage of cleaved caspase-3 positive cells when compared to those glands treated with radiation alone (p<0.001). Cleaved caspase-3 levels were also quantified 48 hours following radiation. Again, a significantly higher percentage of cleaved caspase-3 positive cells is found in tissues treated with radiation alone (p<0.001) albeit lower than levels at 24 hours. Irradiated parotid glands pretreated with Roscovitine show a significantly reduced amount of cleaved caspase-3 positive cells when compared to radiation 48 hours post treatment (p<0.05). Roscovitine alone does not induce apoptosis at either time point, as there is no significant difference in the percentage of cleaved caspase-3 positive cells when compared to untreated levels. These findings suggest that Roscovitine suppresses apoptosis in irradiated salivary glands potentially through the activation of Akt.

### Preserved Salivary Flow Rates in Irradiated Parotid Glands Pretreated with ROSCOVITINE at Acute Time Points

**Figure 3:**
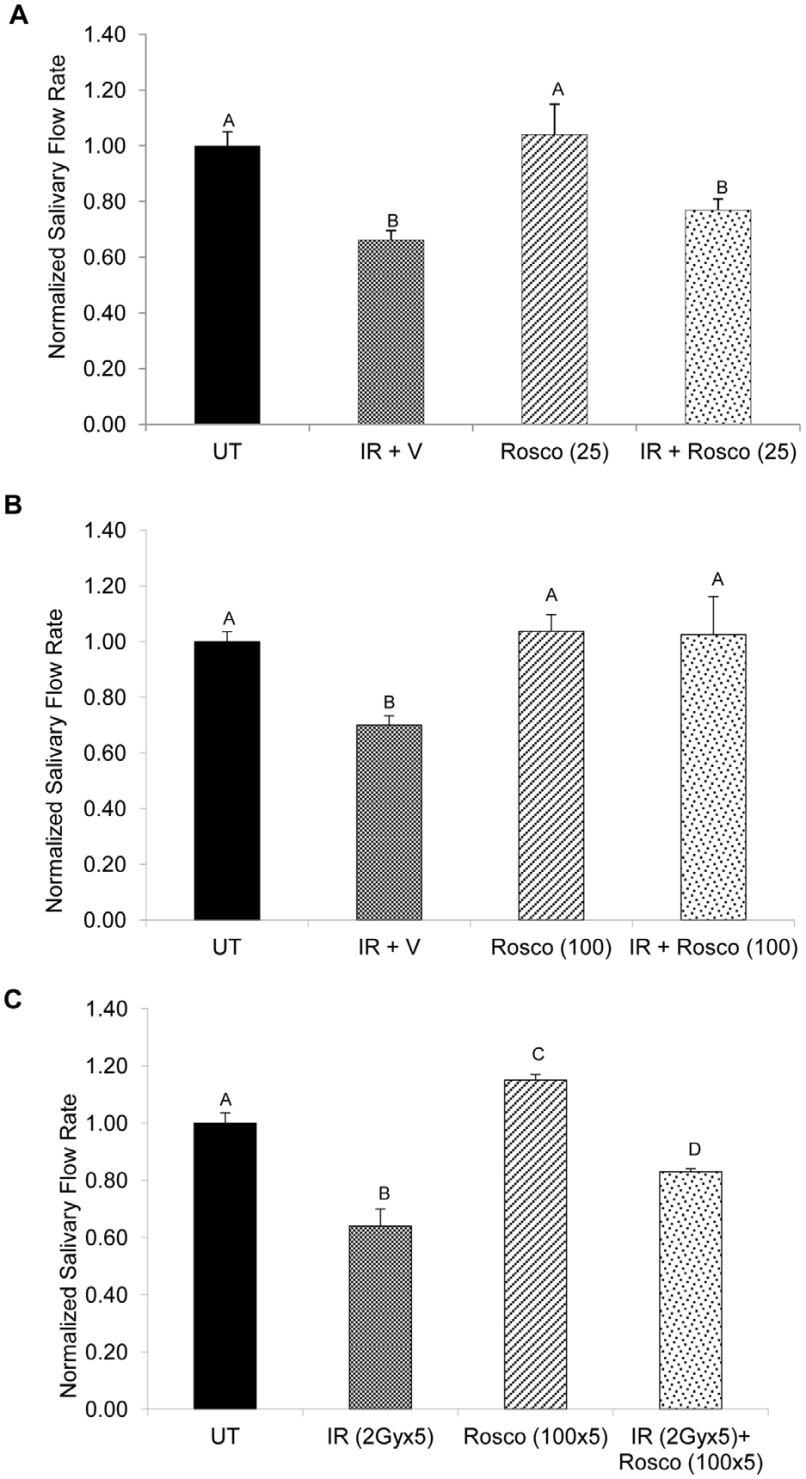
Preserved salivary flow rates in irradiated parotid glands pretreated with Roscovitine at acute time points. Mice were treated as stated in Figure 1. Three days following radiation treatment, stimulated saliva was collected for five minutes immediately following an injection of carbachol. Abbreviations represent untreated (UT), irradiated with vehicle pre-treatment (IR+V), Roscovitine alone (Rosco), and Roscovitine prior to irradiation (IR+Rosco). A) Mice received a dose of 25 mg/kg Roscovitine prior to radiation treatment. B) Mice received a dose of 100 mg/kg Roscovitine prior to radiation treatment. Salivary flow was normalized to unirradiated controls (UT). Results are shown as the mean +/− SEM with at least eight mice per treatment group. C) Mice received a dose of 100 mg/kg Roscovitine prior to 2 Gy radiation treatment for five consecutive days. Salivary flow was collected 14 days after the final radiation treatment and normalized to unirradiated controls (UT). Results are shown as the mean +/− SEM with at least three mice per treatment group. Treatment groups with the same letters are not statistically different from each other.

Previous studies have shown that irradiated salivary glands pretreated with IGF-1 have preserved salivary flow rates at both acute and chronic time points following a single dose of radiation [8], [9]. To show that Roscovitine is able to acutely preserve salivary flow following radiation, stimulated saliva collections were performed 3 days following radiation comparing two doses of Roscovitine (25 mg/kg, Figure 3A or 100 mg/kg, Figure 3B). In mice that received radiation treatment, a significant reduction in salivary flow rates is seen when compared to unirradiated controls (p<0.001). Irradiated mice pretreated with 25 mg/kg Roscovitine have a small improvement in salivary flow rate that was not statistically different than radiation treatment (77% vs. 66% of control levels). In contrast, irradiated mice pretreated with 100 mg/kg Roscovitine have significantly higher salivary flow rates when compared to radiation (p<0.01) and are not statistically different from unirradiated controls. Mice treated with either 25 mg/kg or 100 mg/kg Roscovitine alone show salivary flow rates similar to unirradiated controls. Patients with head and neck cancer are treated with a fractionated radiation regimen with salivary gland exposures at ∼2 Gy/day [4]. To test the effects of Roscovitine under a more clinically relevant fractionation scheme, mice were treated with 2 Gy/day for five consecutive days resulting in a cumulative dose of 10 Gy and stimulated saliva collections were performed 14 days after the final radiation treatment (Figure 3C). Mice exposed to fractionated radiation have significantly reduced salivary flow rates when compared to unirradiated controls (p<0.001). In contrast, mice receiving 100 mg/kg Roscovitine prior to fractionated radiation have significantly improved salivary flow rates when compared to irradiated animals (p<0.01). These results suggest that pretreatment with Roscovitine can preserve normal salivary gland physiology at acute time points following radiation.

### Preserved Salivary Flow Rates in Irradiated Parotid Glands Pretreated with Roscovitine at Day 30

**Figure 4:**
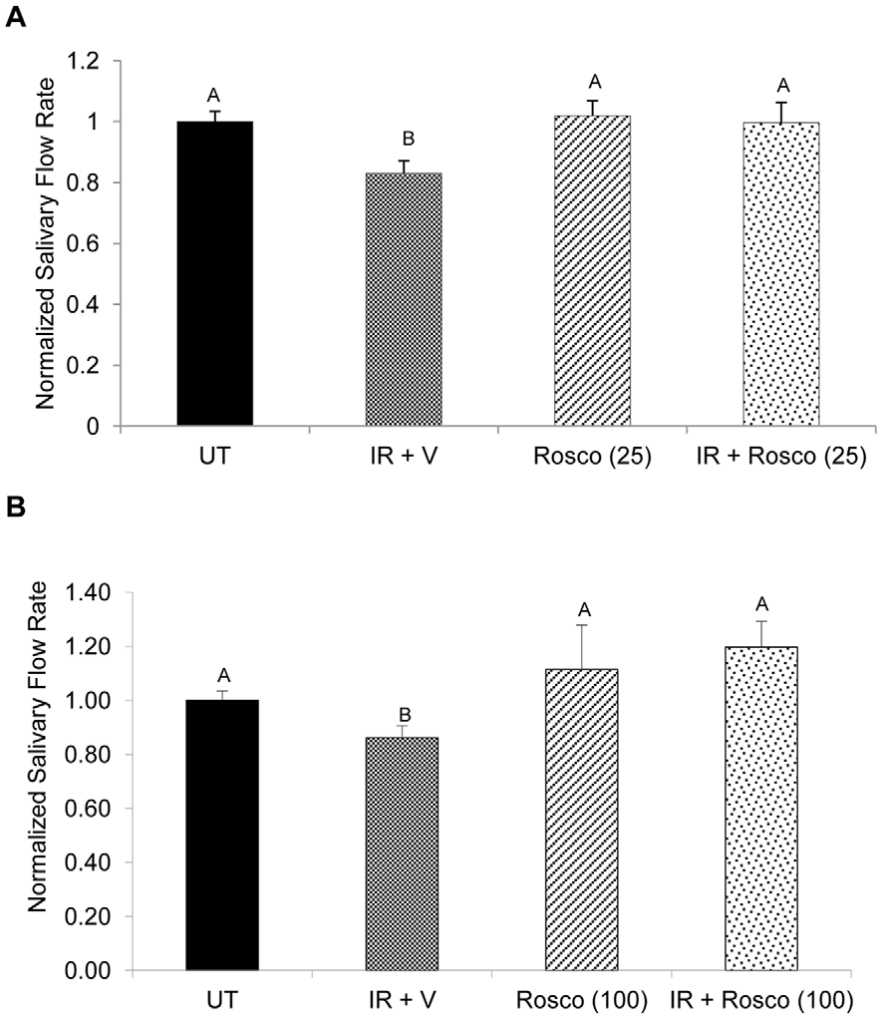
Preserved salivary flow rates in irradiated parotid glands pretreated with Roscovitine at Day 30. Mice were treated as stated in Figure 1. Chronic dysfunction was evaluated thirty days after radiation treatment using stimulated saliva collections for five minutes immediately following an injection of carbachol. Abbreviations represent untreated (UT), irradiated with vehicle pre-treatment (IR+V), Roscovitine alone (Rosco), and Roscovitine prior to irradiation (IR+Rosco). A) Mice received a dose of 25 mg/kg prior to radiation treatment. B) Mice received a dose of 100 mg/kg prior to radiation treatment. Salivary flow was normalized to untreated. Results are shown as the mean +/− SEM with at least eight mice per treatment group. Treatment groups with the same letters are not statistically different from each other.

To observe the long-term chronic effects of Roscovitine on preservation of salivary flow rates, stimulated saliva collections were performed 30 days following a single dose of 5 Gy radiation (25 mg/kg, Figure 4A or 100 mg/kg, Figure 4B). Mice treated with radiation continue to show a significant reduction in salivary flow rates when compared to unirradiated controls (p<0.05). In irradiated mice pretreated with 25 mg/kg Roscovitine, salivary flow rates return to levels comparable to unirradiated controls. Similarly, mice treated with 100 mg/kg Roscovitine show preserved salivary flow rates that are significantly higher than radiation treatment (p<0.01). In mice treated with Roscovitine alone, at either 25 mg/kg (Figure 4A) or 100 mg/kg (Figure 4B), salivary flow rates are similar to unirradiated controls.

**Figure 5:**
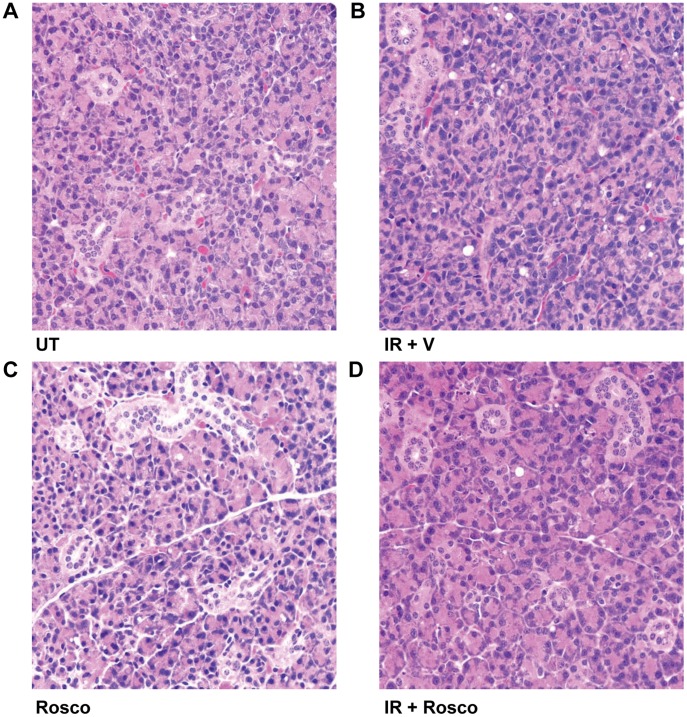
Histological review of salivary glands 30 days following Roscovitine pretreatment in irradiated tissues. A) Untreated parotid gland showing structure of acini and ducts. B) Parotid gland treated with a single dose of 5Gy radiation C) Parotid gland treated with Roscovitine. D) Parotid gland pretreated with Roscovitine thirty days following a single dose of 5Gy radiation. Abbreviations represent untreated (UT), irradiated with vehicle pre-treatment (IR+V), Roscovitine alone (Rosco), and Roscovitine prior to irradiation (IR+Rosco).

Histological review of the parotid salivary glands from each of the treatment groups (unirradiated, irradiated, Roscovitine alone, and Roscovitine prior to irradiation; figure 5) was conducted thirty days following treatment. Radiation-induced fibrosis was not observed in either of the radiation treatment groups. Additionally, few vacuoles were observed in any of the treatment groups. Together, these data suggest that Roscovitine preserves salivary gland function without any detrimental effects on overall structure at chronic time points following radiation.

## Discussion

Patients undergoing radiotherapy for treatment of head and neck cancer often suffer from debilitating side effects related to radiation-induced damage of the salivary glands and loss of stimulated salivary flow. The current palliative therapies for relieving the side effects of salivary gland dysfunction are short lived and often are accompanied by their own unwanted side effects. In this study we focused on the use of Roscovitine, a cyclin-dependent kinase inhibitor, as a possible preventative therapy for salivary gland dysfunction. We found that Roscovitine improved salivary flow rates in irradiated mice at both early and late time points. Importantly, these improvements were not statistically different from unirradiated controls suggesting a complete preservation of normal tissue function. This preservation of salivary physiology by Roscovitine is similar to previous work utilizing IGF-1 pretreatment [8].

The majority of investigations with Roscovitine and its derivatives have examined its effects on cancer cell lines and xenograft models. Since Roscovitine was developed as a cdk inhibitor, initial work focused on its effects on cell cycle progression [11]. Subsequent studies using a combination of Roscovitine and radiation have reported minimal increases in G**_2_**/M arrest over radiation alone [18], [19]. Peptide inhibitors that disrupt the phosphorylation of downstream substrates of cdk2 (e.g. Rb) have been demonstrated to induce cell death [20], [21]. Similar to the peptide inhibitors, Roscovitine enhanced radiation-induced apoptotic death of cancer cells and markedly reduced tumor growth in vivo [18], [19], [22]. In contrast to these studies, this work focused on the *in vivo* effects of Roscovitine in combination with radiation on normal salivary glands. We found that Roscovitine administered prior to radiation induced a significant increase in the accumulation of cells in the G**_2_**/M phase of the cell cycle. This induction of cell cycle arrest leads to a decrease in the level of apoptosis in irradiated tissues pretreated with Roscovitine. These results are similar to the peptide inhibitor studies where cell viability was unaltered by treatment of non-transformed normal cells [20], [21]. We hypothesize that one mechanism of salivary gland sensitivity to radiation involves inefficient cell cycle arrest leading to poor DNA repair and elevated levels of apoptosis [10]. The current study supports the notion that transient activation of cell cycle arrest in normal tissues can have a beneficial effect in preserving cell viability and function.

Radiation causes DNA damage, which activates p53 leading to cell death or cell cycle arrest and DNA repair. It has previously been documented that radiation-induced apoptosis of acinar cells is regulated by p53 [23]. In addition, IGF-1 mediated cell cycle arrest following radiation is p53-dependent. Therefore, protection of normal tissues from radiation-damage may involve directing p53 activity toward cell cycle arrest rather than apoptosis. Our previous work has demonstrated that the p53 ubiquitin ligase, MDM2, is required for Akt-mediated suppression of apoptosis [24] and treatment with IGF-1 prior to radiation enhances p53 binding to the p21 promoter leading to sustained expression [10]. In the current study, we observed increased p21 expression and elevated phosphorylation of Akt and MDM2 in salivary gland tissues treated with Roscovitine prior to radiation. Interestingly, Mendoza et al reported that Roscovitine reduced cellular viability in cells with inactivated Rb and p53 function when compared to parental cells with functional Rb and p53 [21]. This suggests that Roscovitine-mediated protection of normal tissues may be dependent on wildtype p53 activity. This is an important distinction between normal tissues and cancer cells, since a number of tumors have mutated or altered p53 activity [6].

Important for the translation of the findings of the current study into the clinic, R-roscovitine (seliciclib) is currently in clinical trials as a small molecule therapeutic for treatment of advanced solid tumors and non-small cell lung carcinomas [25]. In this study, Roscovitine preserved the physiological function of normal salivary glands at acute and chronic time points by arresting the cell cycle at the G**_2_**/M phase and suppressing apoptosis. This is clinically significant because salivary glands have demonstrated little capacity for regeneration and therefore tissues exposed to higher cumulative doses of radiation (>30 Gy) often have permanent loss of function [26]. In addition, this is potentially broadly applicable to other highly differentiated tissues in close proximity to tumor margins. Based on the new knowledge from the current study, Roscovitine has a potential additional use as a small molecule therapeutic in preventing the damage of incidental radiation to surrounding normal tissues like salivary glands. This would be a significant benefit to the quality of life of patients undergoing radiation treatment for head and neck cancer.

## References

[1] Siegel R, Naishadham D, Jemal A (2012) Cancer statistics, CA Cancer J Clin. 62: 10–29. 10.3322/caac.20138 22237781

[2] Jerjes W, Upile T, Radhi H, Petrie A, Abiola J, et al (2012) The effect of tobacco and alcohol and their reduction/cessation on mortality in oral cancer patients: short communication. Head Neck Oncol 4: 6. 22409767 10.1186/1758-3284-4-6 PMC3329636

[3] Braam PM, Terhaard CH, Roesink JM, Raaijmakers CP (2006) Intensity-modulated radiotherapy significantly reduces xerostomia compared with conventional radiotherapy. Int J Radiat Oncol Biol Phys 66: 975–980. 16965864 10.1016/j.ijrobp.2006.06.045

[4] Grundmann O, Mitchell GC, Limesand KH (2009) Sensitivity of salivary glands to radiation: From animal models to therapies. J Dent Res 88: 894–903. 19783796 10.1177/0022034509343143 PMC2882712

[5] Hall E (2000) *Radiobiology for the Radiologist*, 5 edn. Philadelphia: Lippincott, Williams and Wilkins;.

[6] Gudkov AV, Komarova EA (2003) The role of p53 in determining sensitivity to radiotherapy. Nat Rev Cancer 3: 117–129. 12563311 10.1038/nrc992

[7] Limesand KH, Barzen KA, Quissell DO, Anderson SM (2003) Synergistic suppression of apoptosis in salivary acinar cells by IGF1 and EGF. Cell Death Differ 10: 345–355. 12700634 10.1038/sj.cdd.4401153 PMC2885155

[8] Limesand KH, Said S, Anderson SM (2009) Suppression of radiation-induced salivary gland dysfunction by IGF-1. PLoS ONE 4: e4663. 19252741 10.1371/journal.pone.0004663 PMC2646143

[9] Limesand KH, Avila JL, Victory K, Chang H-H, Shin YJ, et al (2010) IGF-1 preserves salivary gland function following fractionated radiation. Int J Radiat Oncol Biol Phys. 78(2): 579–86. 10.1016/j.ijrobp.2010.03.035 PMC2939244 20638195

[10] Mitchell GC, Fillinger JL, Sittadjody S, Avila JL, Burd R, et al (2010) IGF1 activates cell cycle arrest following irradiation by reducing binding of DeltaNp63 to the p21 promoter. Cell Death Dis 1: e50. 21480565 10.1038/cddis.2010.28 PMC2939491

[11] Meijer L (1996) Chemical inhibitors of cyclin-dependent kinases. Trends Cell Biol 6: 393–397. 15157522 10.1016/0962-8924(96)10034-9

[12] Raynaud FI, Whittaker SR, Fischer PM, McClue S, Walton MI, et al (2005) In vitro and in vivo pharmacokinetic-pharmacodynamic relationships for the trisubstituted aminopurine cyclin-dependent kinase inhibitors olomoucine, bohemine and CYC202. Clin Cancer Res 11: 4875–4887. 16000586 10.1158/1078-0432.CCR-04-2264

[13] Livak KJ, Schmittgen TD (2001) Analysis of relative gene expression data using real-time quantitative PCR and the 2(-Delta Delta C(T)) Method. Methods 25: 402–408. 11846609 10.1006/meth.2001.1262

[14] Wallace M, Coates PJ, Wright EG, Ball KL (2001) Differential post-translational modification of the tumour suppressor proteins Rb and p53 modulate the rates of radiation-induced apoptosis in vivo. Oncogene 20: 3597–3608. 11439323 10.1038/sj.onc.1204496

[15] Pawlik TM, Keyomarsi K (2004) Role of cell cycle in mediating sensitivity to radiotherapy. Int J Radiat Oncol Biol Phys 59: 928–942. 15234026 10.1016/j.ijrobp.2004.03.005

[16] Bunz F, Dutriaux A, Lengauer C, Waldman T, Zhou S, et al (1998) Requirement for p53 and p21 to sustain G2 arrest after DNA damage. Science 282: 1497–1501. 9822382 10.1126/science.282.5393.1497

[17] Cmielova J, Rezacova M (2011) p21Cip1/Waf1 protein and its function based on a subcellular localization. J Cell Biochem 112: 3502–3506. 21815189 10.1002/jcb.23296

[18] Maggiorella L, Deutsch E, Frascogna V, Chavaudra N, Jeanson L, et al (2003) Enhancement of radiation response by roscovitine in human breast carcinoma in vitro and in vivo. Cancer Res 63: 2513–2517. 12750274

[19] Hui AB, Yue S, Shi W, Alajez NM, Ito E, et al (2009) Therapeutic efficacy of seliciclib in combination with ionizing radiation for human nasopharyngeal carcinoma. Clin Cancer Res 15: 3716–3724. 19470731 10.1158/1078-0432.CCR-08-2790

[20] Chen YN, Sharma SK, Ramsey TM, Jiang L, Martin MS, et al (1999) Selective killing of transformed cells by cyclin/cyclin-dependent kinase 2 antagonists. Proc Natl Acad Sci U S A 96: 4325–4329. 10200261 10.1073/pnas.96.8.4325 PMC16331

[21] Mendoza N, Fong S, Marsters J, Koeppen H, Schwall R, et al (2003) Selective cyclin-dependent kinase 2/cyclin A antagonists that differ from ATP site inhibitors block tumor growth. Cancer Res 63: 1020–1024. 12615717

[22] Zhang F, Zhang T, Gu ZP, Zhou YA, Han Y, et al (2008) Enhancement of radiosensitivity by roscovitine pretreatment in human non-small cell lung cancer A549 cells. J Radiat Res 49: 541–548. 18728343 10.1269/jrr.08024

[23] Avila JL, Grundmann O, Burd R, Limesand KH (2009) Radiation-induced salivary gland dysfunction results from p53-dependent apoptosis. Int J Radiat Oncol Biol Phys 73: 523–529. 19147016 10.1016/j.ijrobp.2008.09.036 PMC2631421

[24] Limesand KH, Schwertfeger KL, Anderson SM (2006) MDM2 is required for suppression of apoptosis by activated Akt1 in salivary acinar cells. Mol Cell Biol 26: 8840–8856. 16982679 10.1128/MCB.01846-05 PMC1636839

[25] ClinicalTrials.gov. 2012.

[26] Li Y, Taylor JM, Ten Haken RK, Eisbruch A (2007) The impact of dose on parotid salivary recovery in head and neck cancer patients treated with radiation therapy. Int J Radiat Oncol Biol Phys 67: 660–669. 17141973 10.1016/j.ijrobp.2006.09.021 PMC2001308

